# Embryo-like features of induced pluripotent stem cells defy legal and ethical boundaries

**DOI:** 10.3325/cmj.2013.54.589

**Published:** 2013-12

**Authors:** Mianna Meskus, Iñigo de Miguel Beriain

**Affiliations:** 1 University of Helsinki, Department of Social Research (Sociology), Helsinki, Finland *mianna.meskus@helsinki.fi*; 2 UPV/EHU. Inter-University Chair in Law and the Human Genome. Bilbao. Spain. *idemiguelb@yahoo.es*; Stem cell science has undergone major paradigmatic and technological transformations following the successes made in reprogramming mature cells to become pluripotent stem cells; a process which is still under way. This article in a series of articles about bio-objects (1) discusses stem cell science and how the question of ethical panacea can be elaborated in the case of induced pluripotent stem (IPS) cells. The stem cells challenge the boundaries (2), which are at play in deriving, applying, and regulating the use of iPS cells. The recent novelty of reprogramming cells not in vitro but in vivo has implications in the demarcation of totipotency from pluripotency in cellular capacities. This in turn complicates the perspective of iPS cells as an ethical panacea to biomedical research on human biological material.

## The latest promise to regenerative medicine: iPS cells

Ethical discussions on regenerative medicine and, specifically on the promises of stem cell science, have largely focused on controversies introduced by stem cells of embryonic origin. This is due to the fact that human stem cells have been derived from embryos donated by couples undergoing in vitro fertilization (IVF) treatment, from donated oocytes, or from aborted fetuses. In this article, we discuss ethical aspects emergent in stem cell science following the successes made in reprogramming “adult” cells to become pluripotent stem cells, not only in vitro but also in vivo. The generation of iPC cells has been a major scientific event, receiving worldwide public attention through, for example, the Nobel Prize in Physiology or Medicine which was awarded in 2012 to John B. Gurdon and Shinya Yamanaka for their work in the field of cell reprogramming.

Reprogramming cells in vitro has been welcomed as a revolutionary technique that “turns back the developmental clock” of human cell development (3,4). This offers new, even unforeseen possibilities in disease modeling and drug discovery. Much of the excitement surrounding iPS cells in basic research, but also in the pharmaceutical industry, concerns what is called a “disease on a dish” approach. By this approach scientists refer to the process whereby stem cells lines in vitro are used to unravel the genetic mechanisms of disease (5,6).

## Boundary-work on the ethicality of iPS cells

Since its beginning, the “stem cell debate” has been predominantly focused on the treatment of embryos as research subjects, on concern about harm to embryos, concern about the potential exploitation of women for their ova, and concern about harm to respect for human life, fuelled by the possibilities of commodification (7). In the recent history, regenerative medicine seemed to be at an uneasy junction. On the one hand, the possibility of generating human cell lines using embryonic stem cells or cells created via somatic cell nuclear transfer was a solid scientific possibility. Even if experiments had demonstrated that clinical applications based on these cell lines would not materialize in the near future due to, for example, the risk of tumorgenecity, there were high expectations about their applicability in the future. On the other hand, stem cell science was facing the fact that cell lines obtained through these techniques involved several ethical, political, and legal problems.

Given this situation of both heightened scientific expectations and ethical tensions, leading to differing policies and regulations worldwide, the work published by Japanese researchers Kazutoshi Takahashi and Shinya Yamanaka opened a new venue in stem cell research. In brief, they demonstrated that the forced expression of only four transcription factors, Oct3/4, Sox2, c-Myc, and Klf4, was sufficient to convert mouse fibroblast cells into embryonic stem cell-like cells (8). Many subsequent articles then confirmed that the timed expression of these factors can change differentiated cells into iPS cells also with human cells (9).

From the perspective of demarcating ethical from non-ethical practices within stem cell research, the possibility of creating pluripotent stem cells without destroying a human embryo in the process has been welcomed by scientists and bioethicists alike. iPS cells alleviate researchers’ dependency on donated embryos and thus are said to bypass ethical problems with research on embryonic tissue. Indeed, iPS cells have been pictured in scientific journals as well as public media as a panacea to the development of stem cell science and regenerative medicine.

Regarding stem cell research and ethics, the principle of safeguarding the dignity and integrity of the person *vis-à-vis* patentability and thus commercialization of inventions based on human biological material is inscribed into the European patent law. However, the case of iPS cells offers an interesting view into this rationale. A definitive green light to the patentability of products and processes drawing from iPS cells came from the legal framework provided by the European Court of Justice some years ago. On the October 18, 2011 the European Court of Justice ruled on the Oliver Brüstle vs Greenpeace case, stating that within the meaning of Article 6[2][c] of Directive 98/44/EC of the European Parliament “any human ovum after fertilization, any non-fertilized human ovum into which the cell nucleus from a mature human cell has been transplanted and any non-fertilized human ovum whose division and further development have been stimulated by parthenogenesis constitute a ‘human embryo’”, and cannot be patented (10).

The reason was clearly indicated in the same document: “Although those organisms have not, strictly speaking, been the object of fertilisation, due to the effect of the technique used to obtain them they are, as is apparent from the written observations presented to the Court, capable of commencing the process of development of a human being just as an embryo created by fertilisation of an ovum can do so.” This statement involved an immediate ban on patents on stem cells produced not only using surplus embryos from IVF but also via somatic-cell nuclear transfer (SCNT) or via parthenogenesis. Furthermore, the Court interpreted the Directive 98/44/EC to mean that an invention has to be excluded from patentability if it involves the prior destruction of human embryos or their use as base material, whatever the stage at which this takes place.

At the same time, however, the ruling of the European Court of Justice left the door open to patents on iPS cells. The way the Court formulated its ruling provided the possibility to patent pluripotent cell lines or products derived from them. The exclusion from patentability concerned the use of totipotent and pluripotent stem cells, the derival of which involves the destruction of human embryos. Thus, it fixed its definition of totipotency to human embryonic stem (hES) cells. The ruling is based on the presumption that totipotent stem cells cannot be technologically created that is, derived through other means than from embryonic tissue. However, this presumption has become arguable, given the scientific finding in stem cell science we will discuss next.

## Pluripotent or totipotent cells? A new ethical challenge arises from cultivating iPS cells in vivo

Since scientific development is often quite unpredictable, what seems to be a long-term agreement might be challenged sooner than we expect. In September 2013, a Spanish team working in the Oncologic Research National Centre (CNIO) published in *Nature* an article that has some puzzling implications (11). It reported that researchers had managed to cultivate iPS cells in vivo within murine tissue using the same reprogramming technique usually applied to derive iPS cells in vitro. The core finding presented in the article is that in vivo iPS cells are extremely similar to embryonic stem cells. Furthermore, according to the authors they are clearly different from in vitro iPS cells.

Regarding the focus of our essay on ethical boundary-making pertaining to iPS cells, this article by Abad et al (2013) invokes an interesting perspective to the presumption presented above that totipotent stem cells can only be derived from embryos (11). The article states that in mice, in vivo iPS cells present a remarkable capacity to undergo trophectoderm lineage differentiation. This means that they possess an unprecedented capacity to produce embryo-like structures. The researchers conclude that in vivo reprogramming allows the acquisition of totipotency features that are absent in ES cells or in standard in vitro reprogrammed iPS cells (12).

This finding, we suggest, has implications for the demarcation of totipotency from pluripotency in stem cells. What we are facing here is a conceptual problem. It is reasonable to ask, does it make sense to talk about pluripotent stem cells if we are dealing with cell lines with totipotency features; cells that seem to be much more similar to ES cells than to iPS cells produced in vitro*.* If it is ultimately the technique that defines the status of the cell (as in Brüstle vs Greenpeace case), it seems clear that in vivo iPS cells should be considered to be pluripotent cells. But, in doing so, are we committing a nominalist mistake that in the long term might have unanticipated consequences? If these cells include features of totipotency, should they be identified and defined as such? Or, will there arise a need for an intermediate term characterizing those cells whose characteristics land between pluri- and totipotency?

## Conclusion

In her discussion on the difficulties of demonstrating the moral difference between iPS cells, hES cells and embryos, Katrien Devolder (2009) points out that if the human embryo is regarded as morally important by the virtue of its potential to become a human being, then every other cell or group of cells with a similar potential should be assigned equal moral status. If not, it should be admitted that early embryos do not derive their significance from the potential they possess (13). Following this line of thought, our discussion underlines the fact that regarding iPS cells as an ethical panacea to biomedical research and commercialization on human biological material is a narrow perspective to the recent developments in stem cell research and, as such, highly problematic.

Given the plasticity of stem cells, the question of how to define the moral and legal status of iPS cells will become unavoidable, not least because the patent landscape in this field remains largely undefined although it is picking up globally (14). Furthermore, the ethical boundary-making in the case of iPS cells is a topical subject of study, since the cells themselves are bio-objects in-the-making, following the conceptual work in the previous essays of this series (15). As bio-objects of such fluctuating quality, iPS cells point to the observation that boundaries pertaining to human biological material – be they ethical, legal, social, or conceptual – are extremely difficult to keep in the long term. Indeed, the need to re-formulate them seems constant.
